# For Pure Food

**Published:** 1898-06

**Authors:** 


### SELECTIONS.

### FOR PURE FOOD.

THE pure-food bill, from which the following extracts are taken, will prevent the shipping of the products of diseased animals from one State to another.

The enforcement of this act is in the hands of the Secretary of Agriculture, and it may be that the work of the Bureau of Animal Industry will be enlarged and made of still greater value to the country in connection with this measure.

A National Pure-food Congress was held in Washington from March 2d to 5th, to discuss and further this bill. The Congress was made up of representatives of agricultural organizations, State departments, or boards of agriculture and health, trade organizations, etc. There were about four hundred delegates. Among those in attendance were two representatives of the veterinary profession : Dr. D. E. Salmon, of Washington, D. C., and Dr. Leonard Pearson, of Pennsylvania. Certain extracts from the bill follow:

*A Bill for preventing the adulteration, misbranding, and imitation of foods, drugs, and condiments in the District of Columbia and the Territories, and for regulating interstate traffic therein, and for other purposes.*

. . . . . . . . . . .

"That the introduction into any State or Territory or the District of Columbia or foreign country of any article of food, drugs, or condiments which is adulterated or misbranded within the meaning of this act, is hereby prohibited, and any person who shall knowingly ship or deliver for shipment from any State or Territory or the District of Columbia or foreign country to any State or Territory or the District of Columbia, or to a foreign country, or who shall knowingly receive in any State or Territory or the District of Columbia or foreign country, or who, having received, shall knowingly deliver for pay or otherwise, or offer to deliver to any other person, in original unbroken packages any such article so adulterated or misbranded within the meaning of this act, or any person who shall sell or offer for sale in the District of Columbia or the Territories of the United States such adulterated, mixed, misbranded, or imitated foods, condiments, or drugs, shall be guilty of a misdemeanor, and for such offence be fined," etc.

. . . . . . . . . . .

"The term 'food,' as used herein shall include all articles used for food, drink, or condiment by man, whether simple, mixed, or compound."

. . . . . . . . . . .

"That for the purposes of this act an article shall be deemed to be adulterated in the case of food or drink.

. . . . . . . . . . .

"If it consists of the whole or any part of a diseased, filthy, decomposed, or putrid animal or vegetable substance, or any portion of an animal unfit for food, whether manufactured or not, or if it is the product of a diseased animal, or of an animal that has died otherwise than by slaughter.

. . . . . . . . . . .

"That this act shall not be construed to interfere with commerce wholly internal in any State, nor with the exercise of their police powers by the several States."

. . . . . . . . . . .

"That any article of food, condiment, or drug that is adulterated within the meaning of this act and is transported, or is being transported, from one State to another for sale, or if it be sold or offered for sale in the District of Columbia and the Territories of the United States, shall be liable to be proceeded against in any District Court of the United States within the district where the same is found and seized for confiscation, by a process of libel for condemnation. And if such article is condemned as being adulterated, the same shall be disposed of as the said court may direct, and the proceeds thereof, if sold, less the legal costs and charges, shall be paid into the Treasury of the United States. The proceedings in such libel cases shall conform, as near as may be, to proceedings in admiralty, except that either party may demand trial by jury of any issue of fact joined in such case, and all such proceedings shall be at the suit of and in the name of the United States."

